# Adsorption of Lead Ion from Wastewater Using Non-Crystal Hydrated Calcium Silicate Gel

**DOI:** 10.3390/ma14040842

**Published:** 2021-02-10

**Authors:** Shijie Liu, Suping Cui, Hongxia Guo, Yali Wang, Yan Zheng

**Affiliations:** College of Materials Science and Engineering, Beijing University of Technology, Beijing 100124, China; liushijie@emails.bjut.edu.cn (S.L.); hxguo@bjut.edu.cn (H.G.); wangyali1978@bjut.edu.cn (Y.W.); zhengyan2012@emails.bjut.edu.cn (Y.Z.)

**Keywords:** calcium silicate hydrate gel, synthesis, adsorption properties, adsorption stability

## Abstract

In order to obtain low-cost and excellent adsorption materials, this paper used calcium acetate and water glass as raw materials to synthesis hydrated calcium silicate gel by precipitation method. The performance and structure of hydrated calcium silicate gel were systematically studied by X-ray photoelectron spectroscopy, fourier transform infrared spectroscopy, specific surface area analyzer and scanning electron microscope. Studies have shown that, non-crystal hydrated calcium silicate gel (CSH) were successfully prepared, and the removal rate of lead ion using CSH reached more than 90%. The adsorption process is consistent with the pseudo-second-order kinetic model and Langmuir adsorption isotherm model, and the limit adsorption capacity reaches 263.17 mg·g^−1^. The acid treatment experiment proved that the adsorption capacity of lead ion using CSH was satisfactory, and the adsorption rate remained at >60% after 5 cycles. The research may provide a low-cost, high-efficiency and high stability adsorbent.

## 1. Introduction

In recent years, with the rapid development of industries and cities in China, harmful heavy metals such as nickel, cadmium, and lead ions have been discharged into the groundwater, along with mining, metallurgy, chemical, electronics, electroplating and other industries [1,2,3,4], causing serious pollution to the water environment. Since heavy metal ions are not degradable in the water environment, they will pose a serious threat to human health through the enrichment of the food chain [5,6,7,8]. Among them, lead ion cannot be easily removed once they enter the environment. When they enter the body through the respiratory tract and digestive tract, symptoms such as arrhythmia and damage to the immune system will occur, posing a threat to human health [9].

At present, there are many methods to remove lead ion in wastewater, such as, chemical precipitation method [10], redox method [11], ion exchange method [12,13,14], membrane separation method [15], biological method [16], adsorption method [17,18,19,20,21]. Among the above methods for dealing with lead ion pollution, all of the above methods have shortcomings. Chemical precipitation method will produce a large amount of sludge during the treatment process and cause secondary pollution [22]; ion exchange resin used in the ion exchange method has heat resistance and poor recycling [23]; membrane separation method has the disadvantage of high maintenance cost in the later stage [24]. Adsorption method has obvious advantages, such as simple operation, high efficiency, and the adsorbent can be reused [25,26,27,28]. However, some porous adsorption materials are expensive, therefore seeking to prepare new adsorption materials with excellent performance and low price has become a research hotspot of scholars at home and abroad [29,30,31,32,33].

Commonly used adsorbents include silica gel, bentonite, zeolite, activated carbon, graphene and carbon nanotubes [34]. They have unique physical and chemical properties such as porous structure, large specific surface area, abundant adsorption site, which make them have a strong ability to adsorb and remove lead ion, so they are used in different degrees. However, the reasons of preparation cost and adsorption performance limit their wide application. Therefore, it is very necessary to find or develop new low-cost and high-efficiency adsorbents [35]. Calcium silicate hydrate has a wide range of sources and low cost, but there are few studies on its adsorption properties. As a cation exchanger, hydrated calcium silicate (CSH) material can stably solidify heavy metals and radioactive elements in its crystal lattice [36,37]. Zeng Lu et al. studied the influence of hydrothermal synthesis system on the pore structure of CSH gel; through research, they found that the specific surface area of the prepared CSH gel can reach 223.51 cm^3^ g^−1^ [38]. Zhang Hongsen et al. used white mud and oil shale ash as raw materials to prepare calcium silicate hydrate; they found that the maximum adsorption capacity for U^6+^, Pb^2+^, Cd^2+^, Cr^3+^ four heavy metal ions are greater than 200 mg·g^−1^, and the removal rate is greater than 86% [39]. In view of this, this article uses calcium acetate and water glass as raw materials to prepare calcium silicate hydrate gel, analyzes its adsorption performance and adsorption mechanism for lead ion, expands the application of this material. Since the prepared CSH gel is non-crystal and similar in structure to the cement hydration product CSH gel, it is the theoretical basis for the subsequent study of the cement hydration product CSH gel’s adsorption performance for lead ion.

## 2. Materials and Methods

### 2.1. Materials and Equipment

Calcium acetate, water glass (mass fraction is 40%), lead nitrate (Pb(NO_3_)_2_), hydrochloric acid (HCl), sodium hydroxide (NaOH) reagents used are all analytical grade, purchased from Beijing chemical plant (Beijing, China), and deionized water was made by the laboratory.

Experimental and testing equipment mainly includes X-ray diffractometer (XRD-7000, Shimadzu, Kyoto, Japan), specific surface area analyzer (TriStar Ⅱ3020, Micromeritics, Norcross, GA, USA), scanning electron microscope (Hitachi S-3400N, Hitachi, Tokyo, Japan), and inductively coupled plasma emission spectrometer (Optima 7000DV, Perkin Elmer, Waltham, MA, USA)

### 2.2. Method

#### 2.2.1. Sample Preparation

First, calcium acetate was dissolved completely in deionized water by placing it in a triangular flask. After full dissolution and stirring of calcium acetate, water glass solution with a mass fraction of 40% was added to the calcium acetate solution at a constant rate of 1 mL·min^−1^ to control the calcium silicon ratio between 0.8–2.0. The solution was stirred at a temperature of 30 °C and a rotation speed of 600 rpm for 2 h, and the solution was incubated for 3–28 days at constant temperature. Then the product was washed and filtered for many times to remove the remaining impurities on the surface of the product. The sample was dried in a vacuum drying oven (DXL450, Yamato, Tokyo, Japan), and keep the temperature at 60 °C to obtain calcium silicate hydrate gel.

#### 2.2.2. Adsorption Performance

The adsorption performance is carried out in a lead ion solution with a concentration of 50–300 mg·L^−1^, and 0.02 g of CSH was added to the solution. The solution was shaken at 25 °C for 180 min, then centrifuged and the equilibrium concentration was detected. The adsorption capacity and adsorption thermodynamic parameters were calculated.

The removal rate (η) and adsorption amount (Q) of heavy metal ions were calculated by Formula (1) and (2), respectively:

(1) $$Q\;=\;\frac{{(C}_{0}-C_{e})\text{ × }V}{m}$$

(2) $$\eta \;=\;\frac{C_{0}-C_{e}}{C_{0}}\text{ × }100\%$$

In the formula, C_0_ and C_e_ respectively represent the initial concentration and adsorption equilibrium concentration (mg·L^−1^), V represent the solution volume (L) and m represent the adsorbent dosage (mg).

## 3. Results

### 3.1. Structure and Morphology

**Figure 1:**
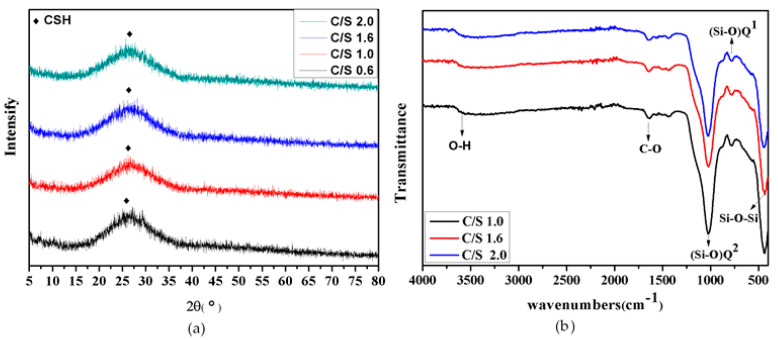
(**a**) Phase of CSH with different calcium silicon ratio, (**b**) infrared spectrum of CSH with different calcium silicon ratio.

In the synthesis process of CSH gel, the main by-product is calcium carbonate. In order to optimize the synthesis process of CSH gel and examine the influence of calcium silicon ratio on the synthesis reaction, Figure 1 shows the XRD of the products under different calcium silicon ratio. As shown in Figure 1a, the diffraction peak at the diffraction angle of 26° is the characteristic diffraction peak of calcium silicate hydrate, and the scattering peak of steamed bread indicating that the CSH gel were all non-crystal substances. It also can be seen from Figure 1b that hydrated calcium silicate prepared under different calcium silicon ratio has (Si–O)Q^1^ at a wavenumber of 790 cm^−1^ and (Si–O)Q^2^ at a wavenumber of 1040 cm^−1^ [40], and the characteristic peak of -OH at the wave number of 3490 cm^−1^, indicating the presence of water molecules in the structure, so it can be considered that the synthesized product is CSH gel. The structure of CSH gel is mainly characterized by its [SiO_4_]^4−^ tetrahedral anion binding state, and the [SiO_4_]^4−^ tetrahedral anion polymerization state will affect the chain length, which will affect the microscopic morphology, specific surface area of CSH [41].

**Figure 2:**
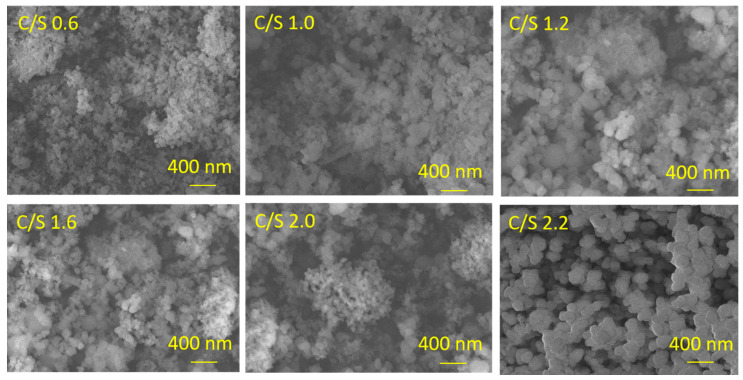
Surface morphology of CSH with different calcium silicon ratio.

CSH with different calcium silicon ratio were prepared by the room temperature precipitation method, and the morphological changes are shown in Figure 2. It can be seen from Figure 2 that when the calcium silicon ratio increases from 0.6 to 1.2, the morphology of CSH particles gradually changes from spherical to dumbbell-shaped, and the particle size gradually increases; when the calcium silicon ratio increases to 1.6, the particle interaction becomes stronger, and agglomeration occurs between the particles; when the calcium silicon ratio increases to 2.0, the gel network structure of CSH gel particles appears, and the particle size decreases; when the calcium silicon ratio is 2.2, the morphology of the CSH gel gradually becomes lamellar. The change of CSH morphology will lead to the change of its specific surface area, pore size and zeta potential, which will affect the adsorption performance of CSH gel to lead ion.

### 3.2. Adsorption Performance of Lead Ions Using CSH

#### 3.2.1. Effect of Calcium Silicon Ratio on the Adsorption of Lead Ions Using CSH

**Figure 3:**
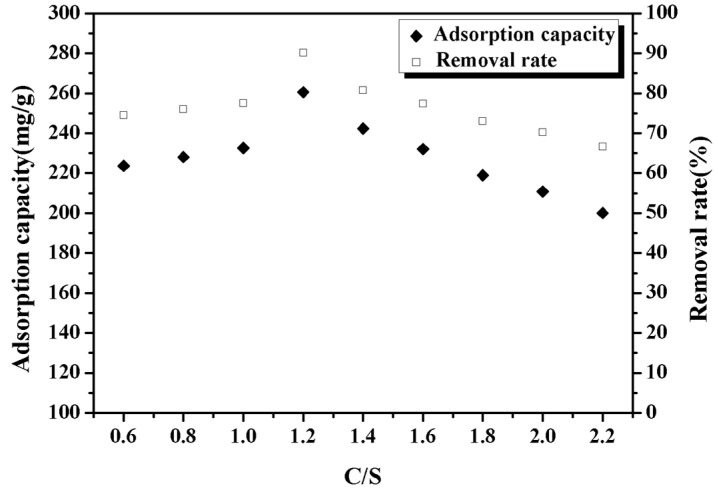
Adsorption of lead ions using CSH with different calcium silicon ratio.

Figure 3 shows the adsorption performance of Pb^2+^ using CSH prepared with different calcium silicon ratio. It can be seen from the figure that the adsorption performance of CSH on Pb^2+^ with different calcium silicon ratio is different. With the increase of calcium silicon ratio, the adsorption performance of CSH on lead ion first increases and then decreases. When the calcium silicon ratio is 1.2, CSH has the best adsorption performance, and the removal rate can reach more than 90%. This may be related to the change in the morphology of the CSH gel caused by different calcium silicon ratio. The morphology change will affect the specific surface area, pore size and zeta potential of the CSH gel. Therefore, we studied the specific surface area, pore size and Zeta potential of the CSH gel with different calcium silicon ratio, the results are shown in Table 1.

According to Table 1, it can be found that the specific surface area of the prepared CSH is between 60 and 130 m^2^·g^−1^, and the zeta potential decreases with the calcium silicon ratio increases. When the calcium silicon ratio is 2.2, the prepared CSH has a large specific surface area of 128 m^2^·g^−1^. The average pore diameter of CSH prepared with different calcium silicon ratio is greater than 2 nm and less than 50 nm, indicating that their pore structures are all mesopores. The pores of this size have strong adsorption capacity and can effectively remove lead ion in wastewater. Appropriate pore size, large specific surface area, large potential difference between CSH and lead ions are beneficial to the adsorption performance.

#### 3.2.2. Influencing Factors of CSH Adsorption of Lead Ions

**Figure 4:**
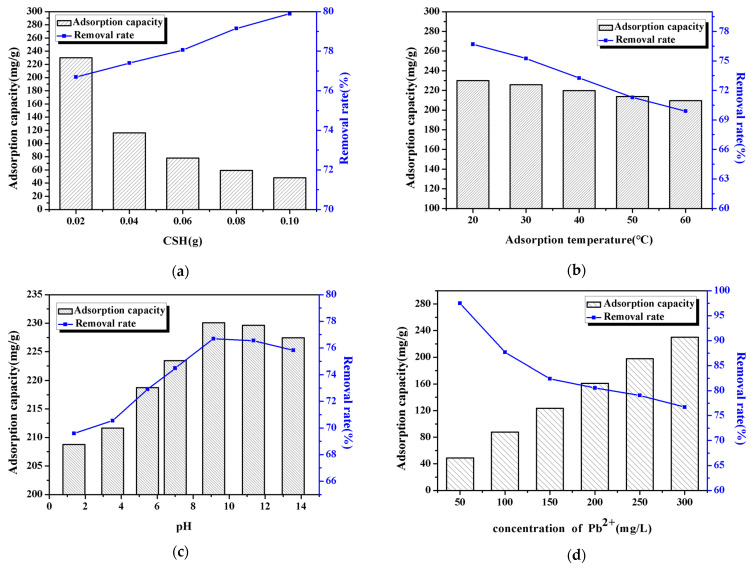
Factors affecting the adsorption of lead ions by CSH: (**a**) CSH dosage, (**b**) adsorption temperature, (**c**) pH value of adsorption solution, (**d**) lead ion concentration.

In order to further study the adsorption performance of CSH on lead ion, we studied the factors affecting its adsorption performance, and the results are shown in Figure 4. It can be seen from Figure 4a that the amount of CSH gradually increased from 0.02 g to 0.1 g, the removal rate of Pb^2+^ increased, while the adsorption amount decreased. This is because when the amount of CSH added to the solution is increasing, the number of active adsorption sites in the system also increases, so the effect of removing lead ion is significantly better; but, at the same time, the content of heavy metal ions in the solution is constant, so the amount of heavy metal ions that can be adsorbed per unit mass of CSH will decrease; according to the calculation formula of the adsorption capacity, the adsorption capacity for the lead ions is reduced. And from Figure 4b, we can see that with the temperature increases, the removal rate and adsorption amount of lead ion by CSH decreases, this is because the adsorption process of lead ions by CSH is exothermic, and temperature rise is not conducive to the adsorption process, therefore, with the temperature increases, the adsorption rate and adsorption capacity of CSH for lead ion both reduced [42].

The pH value will affect the surface properties of the adsorbent. For example, when the pH of the solution is lower than the point of zero charge (PZC) of the adsorbent, the adsorbent surface is positively charged; on the contrary, the adsorbent surface is negatively charged. This will affect the electrostatic force between adsorbent and adsorbate effect [43,44,45]. It was found through experiments that the pH value of the zero charge point (pH_pzc_) of the CSH gel is about 4.0. From Figure 4c, it can be seen that when the pH is less than 4.0, the CSH gel is positively charged and the removal rate of lead ions is low; when the pH is greater than 4.0, the surface is negatively charged, and the removal rate of lead ions increases. When the pH value is greater than 4 and less than 9, the removal rate and adsorption capacity continue to increase; when the pH value is greater than 9, the removal rate and adsorption capacity decrease slightly. This may be because under acidic conditions, the H^+^ in the solution will compete with lead ion for the adsorption site of CSH, which will affect the adsorption effect of CSH on it. When the pH value increases, the H^+^ concentration in the solution decreases and it will reduce the competition between lead ion and H^+^ for adsorption sites, and promote the adsorption of lead ion by CSH. In addition, with the pH value increases, the concentration of OH^-^ in the solution increases, it will react with lead ion to form a hydroxide suspension and precipitate, and the resulting precipitate promotes the removal of lead ion. And it can be seen from Figure 4d that with the increase of the lead ion concentration in the solution, the change trend of the removal rate and the adsorption amount of lead ions by CSH is opposite. In the adsorption process with the initial solubility of 50–300 mg·L^−1^, the removal rate is significantly reduced, while the adsorption capacity increases linearly; before the initial concentration of the solution is 100 mg·L^−1^, the CSH surface structure provides more adsorption active sites, so the removal rate is higher; but when the concentration of the solution increases, the lead ion adsorption active sites that CSH can provide gradually decrease and the adsorption capacity tends to balance, so that the excessive lead ions in the wastewater are not adsorbed, resulting in a decrease in the removal rate.

### 3.3. Thermodynamic and Kinetic Analysis of CSH Adsorption of Lead Ions

**Figure 5:**
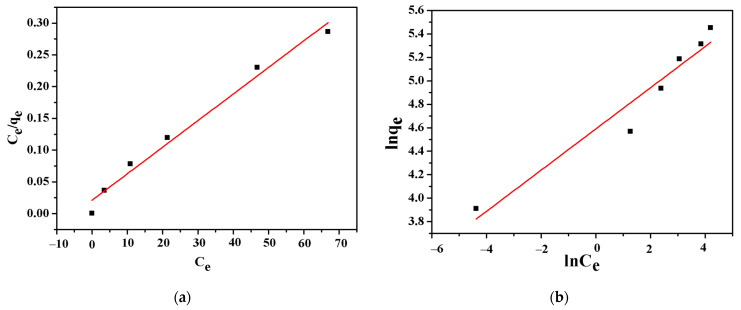
Fitting curves of (**a**) Langmuir and (**b**) Freundlich isotherm models for adsorption of Pb(II) on CSH.

In order to further study the adsorption behavior of CSH on lead ion in wastewater, Langmuir adsorption isotherm model and Freundlich adsorption isotherm model were used to fit the initial concentration of lead ion in the solution with the amount of lead ion adsorbed by CSH. The results are shown in Figure 5, the relevant parameters are shown in Table 2. It can be seen from the correlation coefficient R^2^ in Table 2 that R_L_^2^ > R_F_^2^, which indicates that the adsorption of Pb^2+^ by CSH is more in line with the Langmuir isotherm adsorption model, and the maximum adsorption capacity is 238.10 mg·g^−1^. The adsorption of Pb^2+^ by CSH is similar to that of a monolayer. Adsorption on a uniform surface of the material, its binding sites have the same affinity for adsorption, and the adsorption proceeds uniformly on the surface, which may be due to the chemical interaction between CSH nanoparticles and lead ion.

**Figure 6:**
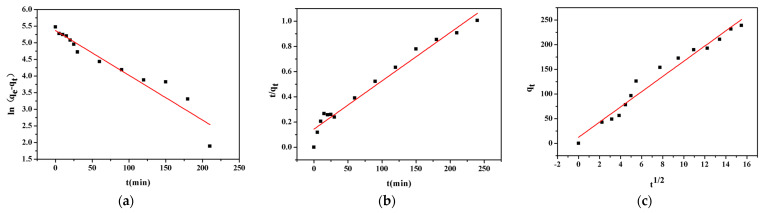
Fitting curves of (**a**) Pseudo-first order and (**b**) Pseudo-second order kinetic models, and (**c**) intra-particle diffusion model for adsorption of Pb(II) on CSH.

In order to better study the principle of adsorption, it is also necessary to analyze the adsorption kinetics. The study of adsorption kinetics is the law of the change of adsorption capacity with time during the adsorption process. It can not only clarify the causal relationship between the structure of the catalytic material and the adsorption performance, but also use the adsorption kinetic equation to analyze the adsorption process and adsorption results. Therefore, the relationship between the adsorption time and the amount of lead ion adsorbed by CSH was subjected to linear regression analysis. The results are shown in Figure 6 and the related parameters obtained are shown in Table 3. The analysis of the correlation coefficient R^2^ in Table 3 shows that the correlation coefficient R^2^ of the pseudo-second order kinetic model is the largest, indicating that the adsorption process of Pb^2+^ by CSH is more in line with the pseudo-second order kinetic model. This is because the pseudo-second order kinetic model includes the entire process of external liquid film diffusion, surface adsorption and intra-particle diffusion, can fully reflect the adsorption mechanism of CSH to lead ion. The degree of fitting of the intra-particle diffusion model is also less than that of the pseudo-second order kinetic model; the adsorption of lead ion by CSH shows a good linear relationship with Q_t_ and t^1/2^, but the equations do not pass through the origin. The adsorption process also includes intra-particle diffusion, but the adsorption process is not the only one, it is also affected by the out-of-particle diffusion. Therefore, it can be concluded that adsorption process of lead ion conforms to the pseudo-second order kinetic model, indicating that CSH has high specific surface area, low internal diffusion resistance, and chemical reaction is the rate-limiting step. It can be said that the most likely mechanism is the ion exchange between CSH and lead ions, which is chemical adsorption [46].

### 3.4. Study on Adsorption Mechanism and Stability of CSH Adsorbing Pb(II)

**Figure 7:**
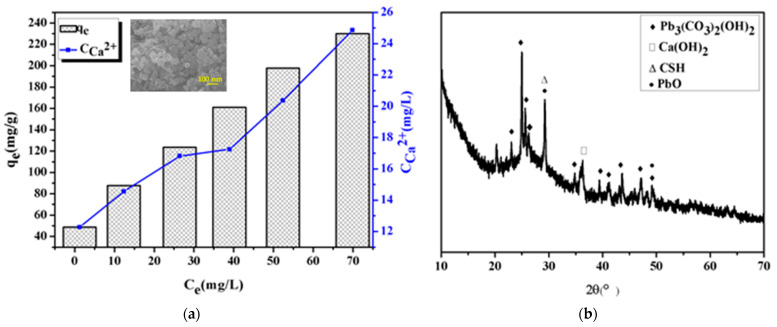
(**a**) Relationship between adsorption amount and Ca^2+^ concentration in solution and SEM image of CSH after Pb(II) adsorption (inset), (**b**) XRD pattern of CSH after Pb(II) adsorption.

The analysis of adsorption kinetics and adsorption isotherms indicated that the adsorption mechanism may be due to the chemical interaction between CSH nanoparticles and lead ion. In order to further study the removal mechanism of lead ion by CSH, the concentration of Ca^2+^ in the solution after adsorption was detected, and the relationship between the adsorption amount and the concentration of Ca^2+^ in the solution was analyzed, as shown in Figure 7b. It was observed that the concentration of Ca^2+^ in the solution increases with the increase of adsorption capacity. And due to Ca^2+^ is a part of the silicon-oxygen tetrahedron in CSH, this may be because Pb^2+^ replaces the position of Ca^2+^. Figure 7 shows the XRD pattern of the adsorbent after the adsorption of lead ion. From the position of the diffraction peak, it can be judged that the products are mainly Pb_3_(CO_3_)_2_(OH)_2_ (PDF No. 01-0687) and PbO (PDF No. 72) 0094). Ca(OH)_2_ is produced by the hydration of CaO, and the generated Ca(OH)_2_ is partially dissociated on the surface of CSH to form OH^-^, therefore, Pb^2+^ in the solution can combine with OH^-^ to form Pb(OH)_2_. Since Pb(OH)_2_ is unstable, it can react with dissolved CO_2_ to form Pb_3_(CO_3_)_2_(OH)_2_; and the existence of PbO can be attributed to the cation exchange between Ca^2+^ and Pb^2+^, which is actually a solid-liquid interface reaction, besides O^2−^ can combine with lead ion to form metal oxides. The chemical reaction that occurs during the adsorption process as follows:

(3) $$\mathrm{CaO}\;+\;H_{2}O\;\to \;Ca{\left(OH\right)}_{2}$$

(4) $$3Pb^{2+}\;+\;Ca{\left(OH\right)}_{2}\;+\;2CO_{2}\;+\;2H_{2}O\;\to \;Pb_{3}{\left(CO_{3}\right)}_{2}{\left(\mathrm{OH}\right)}_{2}\;+\;Ca^{2+}\;+\;4H^{+}$$

(5) $$\mathrm{CaO}\;+\;Pb^{2+}\;\to \;PbO\;+\;Ca^{2+}$$

The surface morphology of the product after the adsorption of lead ions is shown in the inset in Figure 7a, and the product has a large spherical structure. Obviously, the particle size of the product with adsorbed lead ion is larger than that of the sample before adsorption, and the porous structure disappears, which can be attributed to the adsorption of lead ion. Therefore, the mechanism of adsorption of lead ion in wastewater by CSH can be considered to be caused by the ion exchange between Pb^2+^ and Ca^2+^ in the silicon-oxygen tetrahedron, and part of the OH^-^ in the solution reacts with Pb^2+^ to form Pb(OH)_2_. The adsorption of Pb^2+^ in wastewater by CSH is chemical adsorption. After adsorption, new substances are formed, which leads to changes in the form of CSH, and therefore it is difficult to regenerate.

**Figure 8:**
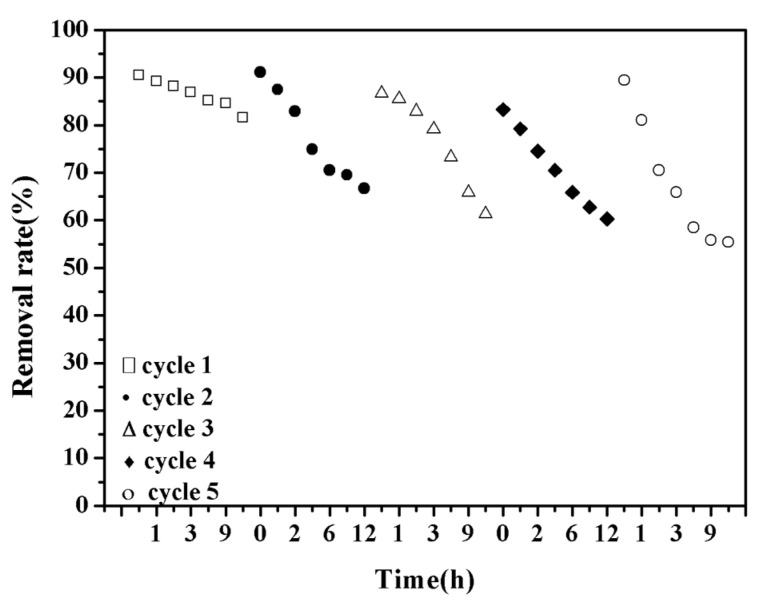
Stability of CSH to adsorb lead ions.

In order to study the stability of the adsorption of lead ion by CSH, a simulated wastewater solution with a concentration of 200 mg·L^−1^ was taken, and the pH value was adjusted to 3.00 with hydrochloric acid. The removal rate is repeated 5 times, and the result is shown in Figure 8. It can be seen from the figure that the adsorption of Pb^2+^ by CSH is relatively stable. In an acidic environment, the removal rate is reduced from 92.36% to 63.52%; the removal rate is still above 60% after 5 cycles. Under acidic conditions, the lead ions adsorbed by the CSH gel will be partially desorbed, this is because the precipitate formed by OH^-^ and Pb^2+^ will dissolve in hydrochloric acid, so the removal rate is reduced. But it is also caused by the ion exchange between the hydroxyl functional groups on the surface of Ca^2+^ and Pb^2+^, the adsorption of Pb^2+^ by CSH gel in wastewater is chemical adsorption, relatively stable new substances will be formed after adsorption, so that the removal rate can still be maintained above 60%.

### 3.5. Comparison with Other Adsorbents

A comparison of lead ion adsorption capacity of the CSH gel to other adsorbents is shown in Table 4. As estimated by the Langmuir model, the maximum adsorption capacity of the CSH gel was 263.17 mg·g^−1^, and the presented data confirm that our adsorbent exhibits higher maximum adsorption capacity than the other previously reported adsorbents. This confirms the excellent application potential of the CSH gel for lead ion adsorption.

## 4. Conclusions

In summary, using low-cost, resource-rich calcium acetate and water glass as raw materials, an non-crystal CSH with a high specific surface area of 128 m^2^·g^−1^ and an average pore diameter of 21.33 nm was prepared by a simple precipitation method. And there is no need to add any reagents during the experiment. The product shows excellent ability to remove lead ions, and the optimal experimental conditions were optimized: the amount of adsorbent was 0.02 g, the solution pH = 9–14, and the adsorption temperature was room temperature. Under this condition, the removal rate of lead ion can reach over 90.80%. The adsorption kinetics and isotherm data of the CSH adsorbent are in good agreement with the pseudo-second order kinetic model and Langmuir model, indicating that lead ion were chemically adsorbed on the magnesium oxide adsorbent in a single layer, and its maximum adsorption capacity is 263.17 mg·g^−1^. We propose an adsorption mechanism involving hydroxyl functional groups and ion exchange between Ca^2+^ and lead ion on the CSH surface. The above results show that the CSH adsorbent has the advantages of high efficiency, easy preparation, easy promotion and harmless to the environment, which is expected to become an excellent adsorbent for the rapid removal of lead ion in wastewater.

## Figures and Tables

**Table 1:** Specific surface area(a_BET_) and zeta potential of CSH gel with different calcium silicon ratio.

Calcium-Silicon Ratio	a_BET_ (m^2^·g^−1^)	Average Pore Size (nm)	pH Value of the System	Zeta Potential (mV)
0.6	68	9.25	9.38	−21.26
1.2	122	11.53	9.45	−19.16
2.0	124	14.54	9.42	−11.32
2.2	128	13.43	9.48	−9.86

**Table 2:** Fitting parameters of Langmuir and Freundlich adsorption models for adsorption of Pb(II) on CSH.

Langmuir Parameter			Freundlich Parameter		
Q_m_ (mg/g)	K_L_ (L/mg)	R_L_^2^	K_F_ (L/mg)	1/n	R_F_^2^
238.095	0.2	0.9835	98.5634	5.6915	0.9433

**Table 3:** Fitting parameter of Pseudo-first order and Pseudo-second order kinetic models and intra-particle diffusion model for adsorption of Pb(II) on CSH.

Pseudo-First Order Kinetic Model			Pseudo-Second Order Kinetic Model			Intraparticle Diffusion Model	
k_1_ /min^−1^	Qe /mg·g^−1^	R^2^	k_2_ /g·mg^−1^·h^−1^	Qe /mg·g^−1^	R^2^	K_id_ /mg·(g·h^0.5^)^−1^	R^2^
0.0172	250.6105	0.8729	0.0001015	263.1679	0.9697	15.419	0.9678

**Table 4:** Maximum Pb(II) adsorption capacities of the CSH gel and other reported adsorbents.

Adsorbents	Qm (mg·g^−1^)	References
Cellulose-MT-CBM ^a^	39.02	[47]
SC/ALG ^b^	179.10	[48]
NCS/SA/MC ^c^	114.47	[49]
CA/PCL ^d^	70.50	[50]
CSH gel	263.17	This work

^a^ Cellulose-metallothionein-carbohydrate-binding module biosorbent; ^b^ Saccharomyces cerevisiae/alginate composites beads; ^c^ Nanochitosan/sodium alginate/microcrystalline cellulose beads; ^d^ Cellulose acetate/polycaprolactone.

## Data Availability

The data presented in this study are available on request from the corresponding author.
